# Dietary and nutritional supplementation strategies for table tennis athletes: a mini-review

**DOI:** 10.3389/fnut.2026.1849900

**Published:** 2026-06-26

**Authors:** Ziyang Wang, Lulu Shen, Song Gao

**Affiliations:** 1 College of Physical Education and Health Sciences, Zhejiang Normal University, Jinhua, China; 2 University Table Tennis Team, Zhejiang Normal University, Jinhua, China; 3 Jinhua Deren Rehabilitation Equipment Corporation, Jinhua, China

**Keywords:** diet, indoor sports, racket sports, recovery, table tennis

## Abstract

Table tennis (TT) has been defined as a fast and explosive aerobic sport. Athletes are required to move expeditiously around the table to catch the ball and produce a strong return. The body utilizes glycogen and fat stores as the primary energy sources. Furthermore, this sport demands that athletes possess not only explosive power, speed and endurance, but also anaerobic capacity. Consequently, these qualities are contingent on a combination of physical, technical and psychological attributes. TT demands a high level of concentration and technical proficiency from athletes, encompassing elements such as precise hitting, rapid responsiveness, and exacting control. This highly concentrated technical requirement will consume a significant amount of energy. A balanced and nutritionally adequate diet is essential for athletes, as it provides the energy and nutrients necessary to support their training and competition requirements. The present review is predicated on the physiological needs of TT, including energy consumption during training and competition, and the main metabolic pathways of TT. This paper provides nutritional suggestions for TT athletes. In addition to the hydration strategy, the daily intake guidelines of carbohydrate, protein and fat are also discussed. Micronutrients, including iron, magnesium and vitamin D, also provide their recommended intake.

## 1 Introduction

Table tennis (TT) is an indoor racket sport that requires players to demonstrate comprehensive qualities of strength, endurance, agility, mental alertness and quick response (1, 2). The objective of each point is to send the ball to the opponent's table, with the intention that the opponent is unable to catch the ball or produce an effective return. TT is the second most popular racket sport worldwide and the most watched sport in China during the 2021 Olympic Games, with an audience of nearly 350 million (3). It is evident that body mass and composition are recognized factors that influence sports performance (4). However, no correlation has been demonstrated between the current survey of athletes' height and weight with their performance (5, 6). TT, like other racket sports, requires high physical strength, and athletes may need to participate in several games in 1 day (singles and doubles or team games), which usually last for 1 week (7). This competition procedure underscores the significance of timely and adequate refueling for optimal physical performance in TT athletes.

A study simulated the metabolic pathway of TT, with 97% (± 2%) of the energy being provided by aerobic respiration, 1% (± 0.7%) by anaerobic respiration, and 3% (± 1%) by phosphocreatine decomposition (8). It is hypothesized that excellent anaerobic ability may be beneficial to more explosive hitting and returning. Consequently, despite the limited proportion of anaerobic pathways, an augmented anaerobic capacity can be sufficient to accommodate high-intensity confrontations (9). Carbohydrates represent the primary energy source for table tennis players, concurrently ensuring adequate creatine intake and its subsequent storage in muscles as saturated creatine phosphate. Nutrients that enhance cognitive acuity and rapid response may result in a reduction of reaction time.

The energy consumption of TT athletes is contingent on their training load and intensity (10). Male TT athletes engage in training regimens that span a duration of 3 h on a daily basis. The estimated daily energy expenditure for these individuals ranges from 3,700 to 4,500 kilocalories (11). The metabolic demand of TT practice ranges from 4.5–5.2 metabolic equivalents (METs) in the absence of footwork to 9.5–11.5 METs with footwork (11). The metabolic equivalents of TT training and simulated competition have been determined to be 8.5 and 10.5 METs, respectively (2, 8, 12). TT is categorized as a moderate-intensity sport. The strategic adjustment of food intake in accordance with energy demand has been demonstrated to facilitate the management of physique among athletes, thereby optimizing their intake to adapt to training and competition. The evaluation of the nutritional adequacy of the diet of elite TT athletes is a field that merits further study in the future.

A proper nutrition strategy has been demonstrated to improve sports performance and promote physical recovery (13). The ingestion of carbohydrates has been demonstrated to enhance endurance, while the consumption of carbohydrates and protein post-exercise has been shown to facilitate recovery (14). However, there is a paucity of research on the special nutrition of TT. It is imperative to be cognizant of the physiological, sports and competition-related needs of TT in order to apply extant sports nutrition knowledge and provide evidence-based recommendations for table tennis. The objective of this review is to evaluate the current physiological demands of TT and to provide nutritional recommendations, including carbohydrates, protein, and selected micronutrients, as well as potential research areas for future studies.

## 2 Methodology

A narrative review was conducted from January 2021 to December 2025. From the beginning to June 2025, English peer-reviewed journal articles were identified through searches of PubMed and Google Academic. The following search terms were utilized: ‘table tennis' and ‘energy metabolism'. The objective of this search was to identify relevant literature on the energy requirements of TT. A subsequent search employs the search terms ‘table tennis' and ‘nutrition' to identify relevant articles concerning this specific group of athletes. A subsequent step in the research process involves conducting a manual search to identify additional references that are pertinent to the subject under discussion. These references may include antioxidants and omega-3 supplements, among others.

## 3 Characteristics of energy metabolism in table tennis

The characteristics of the three energy supply systems are to be combined with the characteristics of TT competition time (see Table 1). TT can be defined as an intermittent sport, comprising a variety of low-intensity activities in the absence of the ball, and a range of high-intensity activities with the ball. It is estimated that the activities in the absence of the ball can account for 30%−60% of the total duration of the sport. In the context of major international competitions, the duration of an athlete's participation can extend to approximately 10 days, which is considered a substantial period. The duration of a round is approximately 7 s. During this period, the athletes are required to perform a series of actions, including the vigorous swinging of the save ball, the rapid movement of the save ball toward the platform, and the pulling of the locked loop ball in the middle and near the platform (15). From a temporal perspective, the energy supply required for seven seconds of rapid explosive movement can only be met by the speed of the ATP-CP energy supply system. The movement form of the TT competition, which is characterized by a sequence of “short load-interval-short load” intervals, results in an average load intensity that is lower than that of other sporting activities. The interval between two rounds is approximately 17 s, whereas the half-time reaction of ATP-CP is 20–30 s. This indicates that ATP-CP has not yet fully recovered from the end of one round to the beginning of the next. Consequently, in the subsequent round of competition, there will be an inadequate ATP-CP energy supply, resulting in the body's activation of the lactic acid energy system.

**Table 1:** Three major energy supply systems.

Energy supply system	substrate	Reserves mmol/kg dry muscle	Synthetic ATP amount mmol/(kg·s) dry muscle	Available exercise time
Phosphate source system	ATP	24.6		6–8 s
	CP	76.8	100	
Glycolytic energy system	Muscle glycogen	365	250	2–3 min
			13,000	1.5–2 h
Oxidation energy system	Fat	48.6	Unlimited	Unlimited
	Protein			

As demonstrated in the preceding analysis, TT is categorized as a moderate-intensity endurance aerobic exercise, with the predominant reliance on the anaerobic energy supply system during periods of intense ball activity. During the interval of no-ball activities, the exercise intensity decreases, with the aerobic energy supply system becoming the predominant source of energy. Consequently, TT players must possess not only a robust aerobic energy supply system, but also an equally developed anaerobic energy supply system. The aerobic energy supply system has been demonstrated to facilitate the restoration of ATP and CP, thereby reducing the accumulation of lactic acid within the body and decelerating the onset of fatigue.

## 4 Nutrition suggestions for table tennis players

### 4.1 Carbohydrates

Carbohydrates are regarded as constituting “high-quality” fuel for high-intensity exercise, which exhibits the advantages of low oxygen consumption and rapid energy supply. Carbohydrates are thus considered an important energy supply source during long-term and high-intensity exercise (16). It is generally recommended that the intake of carbohydrates should be 5 to 8 times body mass to ensure adequate energy supply. Given that TT training and competition primarily constitute aerobic exercise, the recommended carbohydrate intake for table tennis players during the competitive season is 5–7 g/kg body weight per day with this amount varying according to gender, the duration and intensity of exercise, and environmental conditions (17). It is recommended that table tennis players give consideration to the types of carbohydrates they consume. It is imperative that athletes maintain optimal blood glucose levels by consuming nourishment at regular intervals both prior to and during competition. The ingestion of carbohydrates with a high fiber content and a low glycemic index (GI) prior to competition has been demonstrated to assist athletes in maintaining stable blood glucose levels, enhancing sustained energy and alleviating fatigue following physical exertion (18). For a more detailed analysis of this topic, please refer to Table 2, which provides a comprehensive list of practical suggestions and food examples.

**Table 2:** Practical strategies of nutrition suggestions for table tennis athletes.

Nutrients	Practical strategies for achieving recommended intakes
Carbohydrates	a. Ensure an adequate intake of carbohydrate-rich foods (such as rice, noodles, pasta, bread, etc.) b. Choose carbohydrates with a medium to low GI c. Consume carbohydrate-electrolyte drinks during training to maintain blood sugar levels d. Eat foods that contain both carbohydrates and protein (such as milk) to optimize recovery after exercise
Proteins	a. Protein intake should be distributed across three meals and two snacks throughout the day. Recommended sources include milk, lean meat, soy products, and eggs b. The optimal amount of protein per meal is 0.3 g per kilogram of body weight c. Consuming slowly released proteins like casein before bedtime helps promote muscle synthesis and recovery. The protein dose should range from 25 to 40 g
Fat	a. Choose low-fat foods whenever possible, and limit the intake of foods high in saturated fat, such as animal fats b. Polyunsaturated fats (including ω-3 and ω-6 fatty acids) are preferable. These can be obtained from sources like fish, seeds, and nuts
Fluid and water balance	a. Ensure you start exercise in a well-hydrated state b. During high-intensity training or when participating in multiple competitions throughout the day, use carbohydrate-electrolyte drinks to rehydrate
Vitamins and minerals	Iron: Consume red meat or dark green leafy vegetables like spinach Magnesium: Eat plenty of dark green leafy vegetables, whole grains, nuts, and seeds Vitamin D: Regularly check vitamin D levels and consider taking oral supplements as needed. Eat foods rich in vitamin D, such as fatty fish like tuna. Also, get enough sunlight exposure Calcium: Eat dairy products, green leafy vegetables and beans regularly

### 4.2 Protein

The daily consumption of 1.2–2.0 g/kg of protein has been demonstrated to enhance muscle synthesis and repair, thereby improving sports performance (19). TT training and competition demand intermittent sprints, incorporating powerful bursts of movement around the table. Consequently, table tennis players should endeavor to consume protein on a daily basis. Prior to and throughout the competitive season, the recommended daily protein intake for table tennis players is 1.4–1.7 g/kg of body weight, which is analogous to the protein requirements of football and strength training (20, 21). Conversely, during the off-season period, when the training load is minimal, it is recommended that the intake of protein be reduced to 1.2 g/kg of body weight per day. It is recommended that protein be ingested in a dispersed manner throughout the day to ensure optimal absorption (22). It is recommended that athletes consume 0.3 g/kg of protein every 3 to 5 h following training (13). Furthermore, the total daily energy intake must meet the energy consumption required for muscle protein synthesis (22). Athletes should select foods abundant in protein, including lean meat, fish, poultry, beans, dairy products and nuts, in order to facilitate muscle healing and development (13). In instances where the ingestion of protein-rich foods or beverages proves challenging, such as during multi-match days, the use of protein powder can be a viable alternative (23).

### 4.3 Fat

It is imperative to acknowledge the significance of fat in the diet of athletes, as it serves a crucial role in providing energy, along with being a source of essential fatty acids. Additionally, fat functions as a medium for the absorption of fat-soluble vitamins by the body. The World Health Organization (WHO) has set recommended daily intakes for total energy, with a maximum limit of 30% of total energy from fat, and a minimum limit of 10% of total energy from saturated fat (24). The fat intake of TT athletes should conform to the public health guidelines and be individually set according to the training level and body composition (25). It is not recommended for TT athletes to maintain a low fat intake of less than 20% of total energy intake over an extended period, as this may result in a deficiency of essential fat-soluble vitamins and fatty acids (26). It is imperative that foods consumed prior to training or competition be of a reduced fat content, in order to minimize the potential for gastrointestinal discomfort (26).

### 4.4 Liquid and water supplement

Furthermore, athletes should be mindful of replenishing fluids post-exercise to compensate for the loss of fluids and electrolytes during exertion. It is important to note that athletes should be cautious of excessive liquid consumption, as this can lead to hydration and weight gain. It is imperative to maintain optimal hydration levels during physical exertion, as inadequate hydration can compromise exercise performance and elevate physiological strain, particularly in hot environments (26) (see Table 2: Strategies for achieving optimal hydration). During summer months or periods of intense exercise training, TT athletes typically experience elevated levels of perspiration, a consequence of the body's thermoregulatory response to exertion-induced heat generation. Research has demonstrated that even a 1% reduction in body weight due to water deficiency can have a detrimental impact on cognitive function and attention span (27).

In addition to water, perspiration also contains inorganic salts such as sodium, potassium, calcium, magnesium, zinc, copper, and iron, as well as other substances. Consequently, profuse sweating can result in the loss of water and inorganic salts to varying degrees. Disorders in water and salt metabolism have been demonstrated to result in a decline in the body's sports ability, muscle spasms and arrhythmia, which are closely related to sports fatigue, sports injury and sports fever (28). Research has shown that a decrease in body weight by less than 2% to 3% can result in a 30% reduction in athletic ability and an impact on the performance of specific athletic skills (29). It is imperative that, when filling, adherence is observed to the principle of a small amount and many times. Prior to, during and following exercise training, it is advisable to devise a personal hydration plan in conjunction with a sports nutritionist. In addition to ensuring adequate hydration, it is also imperative to consider the supplementation of sodium, potassium, and other trace elements (17, 28). It is imperative to incorporate not only white water, but also electrolytes and sugar, when adding liquid to the mixture. On the day of the competition, when athletes are required to participate in multiple events and/or competitions, it is recommended that they consume carbohydrate electrolyte drinks during periods of rest and after the conclusion of the competition. Athletes can monitor their sweating rate by means of weighing before and after exercise, and adjust their liquid intake plan accordingly.

### 4.5 Key micronutrient requirements

It is imperative to note the pivotal role of vitamins and minerals in immune function, hemoglobin synthesis, and energy metabolism (30). It has been demonstrated that regular exercise can lead to a decline in mineral levels within the body, with magnesium and zinc being notable examples (31). Consequently, athletes may require an increased intake of micronutrients, particularly those engaged in protracted or high-intensity training regimens. It is imperative for TT athletes to adhere to a diet that is characterized by the consumption of a wide variety of foods, with a particular emphasis on fruits and vegetables, in order to ensure that their nutritional intake meets all of their micronutrient requirements. Despite the vital role of B vitamins in energy metabolism, it is improbable that deficiency occurs, given the prevalence of these vitamins in staple foods such as bread and rice (32). Consequently, TT athletes are more concerned about micronutrients such as iron, magnesium, vitamin D and Calcium. The following section outlines strategies to achieve these goals (see Table 2).

#### 4.5.1 Iron

Iron is an essential element in the generation of energy and the formation of oxygen-carrying compounds, as well as hemoglobin and myoglobin. As posited by numerous studies, iron deficiency has the potential to compromise the body's oxygen-carrying capacity, exerting a deleterious effect on the endurance and immune function of athletes (26, 33). However, the results of several surveys indicate that regular aerobic exercise may result in a reduction of iron storage in the body (34, 35). Iron deficiency is a prevalent condition among athletes, particularly among female athletes, vegetarians and frequent blood donors (26). Consequently, TT players who are women, vegetarians or regular blood donors should undergo regular screening to evaluate and monitor their iron status. The objective of TT players should be to consume iron at a slightly higher level than the recommended daily intake.

#### 4.5.2 Magnesium

Magnesium is a cofactor in many enzymatic reactions and plays an important role in anaerobic and aerobic energy production (36). Magnesium has been demonstrated to regulate the cellular movement of calcium, a process that is imperative for the contraction of bones and smooth muscles (36). In individuals with magnesium deficiency, increasing magnesium intake appears to have a beneficial effect on exercise performance, but not in individuals with sufficient magnesium (37, 38). A mounting body of evidence indicates that athletes are not consuming sufficient magnesium from their diet (38, 39).

#### 4.5.3 Vitamin D

Whilst the importance of vitamin D in maintaining optimal bone density and calcium regulation has long been established, recent research indicates that vitamin D also plays a vital role in regulating skeletal muscle function and immune and inflammatory reactions (40, 41). The vitamin D status of athletes has the potential to influence their physical fitness. Ward and colleagues (42) found that vitamin D levels were related to jumping height, speed and strength in young girls after menarche. Currently, there is a paucity of global data on the prevalence of vitamin D deficiency in TT players. In view of the importance of vitamin D to health and muscle function, it is recommended that TT players consider regular blood tests to evaluate their vitamin D status and correct any possible deficiency.

#### 4.5.4 Calcium

Calcium is a mineral that is typically obtained from various dietary sources, including dairy products, green leafy vegetables and beans (43, 44). It has been established that approximately 99% of calcium is stored in the skeletal system, with the remainder existing in muscle cells and other parts (45). It has been posited by Williams (46) and Crede (47) that calcium supplementation may be of benefit to athletes who exhibit inadequate dietary intake. A primary function of calcium is to facilitate the contraction of skeletal muscle tissue (48). Calcium has been demonstrated to assist in the preservation of bone mass in athletes who are predisposed to early-onset osteoporosis, as well as to enhance sporting performance in those with calcium deficiency (47). The bone mineral density of TT players is closely related to their nutrient intake. Maintaining adequate nutrition intake is crucial for preserving and enhancing bone mineral density, thereby optimizing sports performance and mitigating the risk of sports-related injuries.

### 4.6 Dietary supplements

#### 4.6.1 Creatine

Creatine is a compound derived from amino acids methionine, glycine and arginine, which mainly exists in skeletal muscle (49). Most skeletal muscle creatine exists in the form of phosphocreatine (PCr). Supplementation can increase the storage of intramuscular PCr, thus improving the ability of high-intensity exercise and increasing the rate of PCr re-synthesis during recovery (50). Creatine supplementation has been proved to delay fatigue in repeated sprints, increase lean meat, increase the maximum output power of elite athletes and improve post-exercise recovery (51, 52). Creatine supplements have the potential to delay fatigue during competition by enhancing the biosynthesis of PCr (53). TT players may benefit from acute creatine supplementation before competition, such as anxiety or lack of sleep before competition.

#### 4.6.2 Beta-alanine

Beta-alanine is a non-proteinogenic amino acid that is endogenously produced in the liver and can also be obtained through the consumption of meat and poultry (54). Beta-alanine has been demonstrated to enhance the performance of high-intensity exercise, particularly in exercises of less than 60 s, and to reduce neuromuscular fatigue in both male and female subjects (55, 56). However, sensory abnormalities have been reported most frequently as a side effect of beta-alanine use, typically occurring at single doses of 800 milligrams or more (57, 58). In this regard, typical beta-alanine supplementation regimens involve the division of the total daily dose (usually 6–7 grams) into smaller doses (typically 1.4–1.6 grams per dose) to mitigate the sensory abnormalities associated with beta-alanine use (57, 59).

#### 4.6.3 Nitrate

In recent years, nitrate has become increasingly prevalent in dietary regimens, owing to the findings of numerous peer-reviewed studies that have demonstrated its effectiveness in enhancing endurance (60, 61). Nitrate has been found to be present in leafy vegetables such as spinach, lettuce and celery, as well as root vegetables including beetroot (60). Nitrate has been demonstrated to enhance exercise capacity and reduce oxygen consumption during aerobic exercise by increasing blood flow and muscle contractility (62, 63). Researchers frequently administer supplements 2–3 h prior to exercise in order to ascertain the effects of acute dietary nitrate intake. However, the dietary nitrate intake that has been utilized thus far has been in the form of a preventive supplement over a period of 3–6 days (60). However, there is limited information available regarding the time of acute nitrate intake.

#### 4.6.4 Caffeine

Caffeine is a naturally occurring ingredient that is found in certain plants. It is typically ingested through the consumption of beverages such as coffee, tea, cola drinks and energy drinks (64). It has been demonstrated that the substance under scrutiny can enhance a variety of sporting performances, encompassing endurance, high-intensity team sports, strength performance and mental vigilance (64). Consequently, caffeine may have a significant impact on exercises that require high levels of attention, reaction time and technical/tactical skills, which are crucial determinants of physical and mental performance (65). Caffeine has been posited as a potential ergogenic aid for table tennis players, given the sport's emphasis on rapid response times and high velocity. It is anticipated that athletes will participate in numerous games on a daily basis, particularly in instances where they are engaged in multiple events. Evidence has demonstrated that caffeine can enhance performance in simulated table tennis competitions when administered over several days (66). Given the protracted nature of TT matches, which can extend over several days, athletes may consider the ingestion of caffeine at the conclusion of match week in order to enhance or preserve their performance. It is important to note that individuals vary in their reactions to caffeine, and it is therefore essential for athletes to determine their optimal dose prior to competition (67).

#### 4.6.5 Antioxidants

Exogenous antioxidants encompass a wide range of substances, including vitamins, minerals, flavonoids, anthocyanins, tocopherols, polyphenols and carotenoids. These can be obtained from whole foods or dietary supplements (68). Antioxidants are important because they can scavenge free radicals, but also because they participate in the process of controlling oxidative damage and inflammatory reaction in the body. This helps the body to better establish its defense ability (69). While certain dietary antioxidants may offer benefits in reducing muscle fatigue and facilitating recovery in the short term, the long-term and large-dose supplementation of these antioxidants may not yield additional benefits and may, in fact, impede training adaptation (70). The significance of antioxidant supplements is contingent on a number of factors, including the specific objectives of the supplement, the type of supplement in question, the duration and frequency of its use, and the presence of nutritional deficiencies and the individual's basic diet.

#### 4.6.6 Omega-3 fatty acids

Polyunsaturated fatty acids, most notably omega-3 fatty acids such as eicosapentaenoic acid (EPA) and docosahexaenoic acid (DHA), have been demonstrated to play a pivotal role in maintaining redox homeostasis and protecting cells from damage caused by reactive oxygen species (ROS) and reactive nitrogen species (RNS). In contrast to saturated fats, polyunsaturated fatty acids possess multiple carbon double bonds, a structural feature that enhances the fluidity and stability of biological membranes. This, in turn, has been demonstrated to improve the defense against free radicals (71, 72). A substantial body of evidence from randomized clinical trials has been repeatedly demonstrated to demonstrate the efficacy of anti-oxidation and anti-inflammation measures. For instance, Ghiasvand and his colleagues (73) discovered that when EPA was administered at a dose of 2,000 mg per day for a period of 6 weeks, in conjunction with vitamin E supplementation, it resulted in an augmentation in the production of anti-inflammatory cytokines, a diminution in the release of TNF-a, and an additional effect on malondialdehyde. In addition, clinical trials have demonstrated that following long-term supplementation with Omega-3, there is a decline in the concentration of malondialdehyde in serum and/or plasma. The daily intake of a fish oil supplement containing a combination of EPA and DHA, in a quantity of at least 1,440 mg, has been found to be effective (74, 75). In the context of physical exertion, Omega-3 has been demonstrated to possess anti-inflammatory and antioxidant properties, which have the potential to mitigate muscle injury following exercise (76).

## 5 Specific nutrition strategies

The energy expended during physical exertion is replenished and recovered, thereby enhancing and sustaining physical performance over an extended period, and postponing the onset of fatigue. A few days prior to the game, it is recommended that TT athletes consume foods rich in carbohydrates to ensure adequate glycogen storage.

It is customary for TT athletes to consume a light meal 3 h prior to competition, followed by the consumption of light refreshments 1 h prior to the commencement of play, with the objective of ensuring optimal glycemic levels. In order to circumvent the occurrence of gastrointestinal distress, it is imperative that the dietary regimen is characterized by a low fiber and fat content. It is imperative to engage in meticulous planning and utilize familiar comestibles. It is recommended that, prior to a match, players consume a meal that is both simple and easily digestible. Carbohydrates such as bananas, oatmeal bars, and bread are suitable options, as are high-quality proteins found in low-fat milk and eggs.

It is important to note that during the course of the game, players may be required to participate in numerous games within a single day, for a duration that may extend over several days. It is recommended that athletes participating in multiple competitions on the same day ingest carbohydrate beverages or gels to ensure the maintenance of sports skills and to alleviate the cognitive decline caused by fatigue (77). The purpose of restoring nutrition is twofold: firstly, to replenish nutrients and fluids that are lost during exercise, and secondly, to optimize the physical preparation of the body before the next round of exercise. It is imperative to monitor the intake of sugar and electrolytes during the competition. Prior to the event, it is recommended to consume 300 ml of sports drinks 2 h prior to the commencement of the competition. During the event, it is advisable to consume a small amount of fluid immediately following any interruption and to replenish fluids at every break. The cornerstone of nutritional recovery encompasses the restoration of glycogen storage in muscle and liver tissue, the replenishment of sweat losses in terms of liquid and electrolytes, the promotion of protein synthesis in muscle repair and adaptation, and the alleviation of the adverse effects of exercise, including immune dysfunction and inflammatory responses (78).

Inadequate physical rehabilitation following sporting activities can result in inadequate energy provision for subsequent training, thereby impeding the healing process of injuries. The ingestion of carbohydrate-rich foods (sports drinks, bananas, bread, cakes and fruits, etc.) in close temporal proximity to physical exertion has been demonstrated to rapidly replenish muscle glycogen levels and ensure sufficient reserves for subsequent training competitions. In the context of overnight recovery, it is recommended that players consider consuming a meal or snack that is rich in protein prior to retiring for the night. The recommended daily intake of protein for optimal recovery is between 25 and 40 g, which is designed to maximize the synthesis and recovery of protein during the nocturnal period (79). Concurrently, it is recommended to supplement nutrients such as taurine and oligopeptide, which have been shown to facilitate the rapid elimination of fatigue from the muscular system. In the event of athletes participating in multiple games within a short timeframe, the post-game nutritional regime assumes greater significance. The food consumed after a game can be utilized as a source of nourishment until the subsequent meal. It is recommended that athletes consume substantial quantities of carbohydrate-rich foodstuffs during their evening repast. Furthermore, the presence of certain trace elements has been demonstrated to enhance the body's metabolic capacity. This includes iron, zinc, manganese and other trace elements, which have been shown to delay fatigue time and facilitate recovery. Consequently, athletes are able to maintain a high level of competitive ability and performance. It is imperative to meticulously monitor the dietary intake of athletes and ensure the provision of essential nutrients that are lacking in the diets of TT athletes.

## 6 Discussion and conclusion

Nutrition has been demonstrated to have a significant impact on the performance of athletes engaging in racket sports. This impact may be observed in the provision of adequate energy reserves during training, which is a crucial aspect of sporting success. There is a paucity of research on the subject of nutrition in table tennis; however, some evidence can be inferred from research on tennis (80, 81). In order to ascertain the nutritional requirements of table tennis, it is necessary to consider the heat requirement of competition, which can be routinely evaluated by indirect calorimetry (8). The calorie consumption of table tennis (4.6 kcal/min) is higher than that of laboratory work (1.4–2.3 kcal/min), but lower than that of cycling (3.66.0 kcal/min) and running (5.2–7.5 kcal/min) (82). When compared with other racket sports, the heat requirement of table tennis (29-42 kJ/min) is similar to that of badminton (29–46 kJ/min) and tennis (29–46 kJ/min), but lower than that of squash (42–76 kJ/min).

It is recommended that athletes consume a minimum of 55% of their total energy intake in the form of carbohydrates, which is equivalent to 6–10 grams per kilogram of body weight per day. The ingestion of carbohydrates prior to participation in table tennis has been demonstrated to exert a favorable influence on performance, a phenomenon that has been observed to a lesser extent in badminton, where the same nutritional strategy has been shown to enhance the precision of serves (83). Despite the reduced duration of table tennis in comparison to tennis, a similar advantage is observed in terms of carbohydrate intake and the maintenance of blood sugar levels (81).

Due to the fact that table tennis training and competition are conducted within buildings, the indoor temperature and humidity are subject to greater control than in outdoor environments. Consequently, table tennis players may also be affected by dehydration and high temperature. It is evident that elevated ambient temperatures, reduced fluid intake and the undertaking of physical exertion, such as playing tennis, in such conditions can result in an acceleration of the heart rate, an increase in rectal temperature, fluid loss, spasmodic activity and heatstroke. For tennis players, adequate hydration and balanced diet intake between matches have been shown to help maintain physical fitness, even in continuous matches (80). In relation to nutritional habits, the majority of table tennis players' coaches and doctors did not inform them about nutritional supplements. Furthermore, the risk of stimulant use increases with the enhancement of performance level (84).

It is evident that a combination of appropriate nutrition and adequate dietary balance can effectively alleviate the issue of inadequate energy levels experienced by athletes. This, in turn, has been shown to expedite the recovery process from fatigue and enhance their competitive performance. In accordance with the prevailing comprehension of table tennis demand, the field of nutrition has the potential to optimize performance. It is the responsibility of sports nutrition experts to collaborate closely with athletes and coaches to ensure that athletes receive the correct amount and type of nutrients during training and competition.
